# A rare case of locally advanced fibrosarcoma of diaphysal humerus managed successfully with limb-sparing procedures after neoadjuvant chemotherapy

**DOI:** 10.1186/1477-7819-8-77

**Published:** 2010-09-06

**Authors:** Omar El Mesbahi, Samia Arifi, Zineb Benbrahim, Abdelhalim El Ibrahimi, Fouad Kettani, Amal Bennani, Afaf Amarti, My Youssef Alaoui Lamrani, Siham Tizniti, Abdelmajid El Mrini

**Affiliations:** 1 Department of Medical Oncology, Hassan II University Hospital, Route Sidi Hrazem, Fez, 30000, Morocco; 2 Department of Traumatology, Hassan II University Hospital, Route Sidi Hrazem, Fez, 30000, Morocco; 3 Laboratory of Pathology, Avenue Nations Unies, Rabat, 10000, Morocco; 4 Department of Pathology, Hassan II University Hospital, Route Sidi Hrazem, Fez, 30000, Morocco; 5 Department of Radiology, Hassan II University Hospital, Route Sidi Hrazem, Fez, 30000, Morocco

## Abstract

Fibrosarcomas (FS) of bone are a rare malignancy accounting for less than 5% of all primary malignant bone neoplasms. Diagnosis and treatment approaches of this entity are complex and require a skilled and experienced multidisciplinary team.

Authors report their experience with a case of FS of humerus showing a pathologic complete response to neo-adjuvant chemotherapy based on adriamycin, cisplatin and ifosfamid. This approach allowed limb-sparing surgery with an excellent functional and psychological result.

## Background

Fibrosarcomas (FS) of bone represent 5% of all primary bone sarcomas [1,2]. It occurs most frequently in the middle age [1], and affects men and women with equal frequency. In addition, it affects most commonly the long bones. The tumor may be either central (68%) or cortical (31%) [1]. Fibrosarcomas can arises as a primary lesions, or secondary to fibrous dysplasia, to Paget's disease, to bone infarcts, to osteomyelitis, or to post-irradiation of bone and giant cell tumors (GCT) [3,4]. Histologically tumors are characterized by interlacing bundles of collagen fibers without any osteoid, or cartilage production [1]. Differential diagnosis includes fibroblastic osteosarcoma and malignant fibrous histiocytoma (MFH) [5].

Surgery is the standard treatment for fibrosarcoma of bone [1]. Amputation was the primary method of therapy, yielding the best curative results [1]. However, by combining advanced bone imaging techniques with surgical techniques, implant development and neo-adjuvant therapy, limb-sparing surgery can be safely performed.

The role of systemic chemotherapy is not established. Few published reports of chemotherapy in FS of bone have been reported. Chemotherapy regimens used are similar to those used for osteosarcoma. Given to the rarity and heterogeneity of published reports it is not possible to draw conclusions about the role of neo-adjuvant chemotherapy in improving patients outcome and survival.

We present here a rare case of locally advanced FS of bone experiencing complete pathologic response to adriamycin-cisplatin-ifosfamid neo-adjuvant chemotherapy, allowing limb sparing surgery, to illustrate the antitumor activity of this regimen in this rare tumor.

## Case presentation

A 28-year-old woman complained of pain and tumefaction in the upper portion of her right arm. She did not have fever, or trauma. Physical examination, showed a raised mass in the proximal portion of the right arm; with no clinical signs of neurovascular damage. There was no local erythema or skin lesions, and no palpable lymphadenopathy. There were pain and limitation of abduction, internal and external rotation of the right limb.

**Figure 1:**
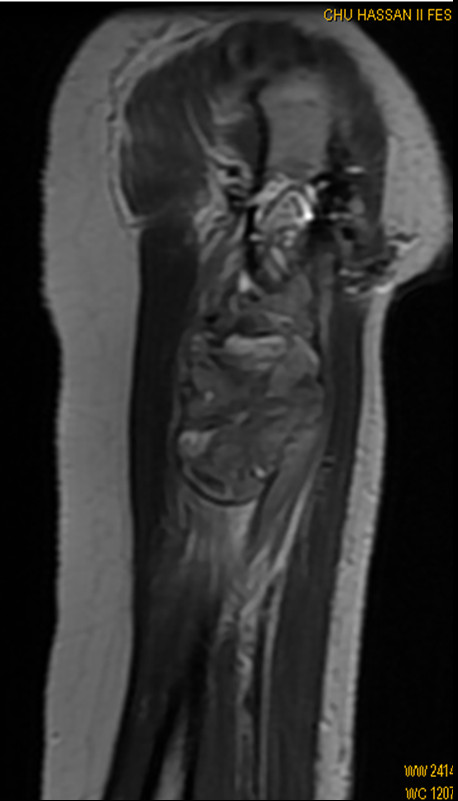
**Sagittal T1 - weighted MR image at the time of first presentation, reveals a huge intra- and extraosseous tumor of the proximal humeral diaphysis**.

The remainder of the physical examination was normal. Radiographs revealed a diaphyseal pathologic fracture involving the right humerus with a periosteal reaction. Magnetic resonance imaging (MRI) of the right arm (Figure 1) showed an eccentric mass in the diaphysis of the humerus, accompanied by an overlying periosteal reaction. The mass extended to a height of 11 cm, and into surrounding soft tissues. The shoulder and elbow appeared normal. There was no skip metastasis.

**Figure 2:**
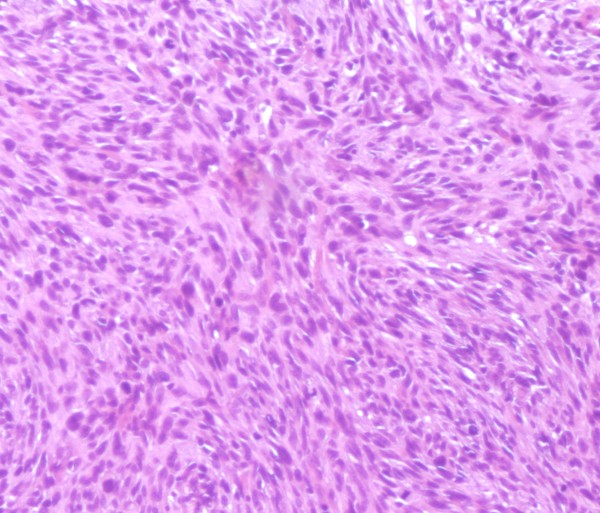
**HESX4 - Spindle cells with interlacing bundles of collagen fibers without any osteoid, or cartilage production**.

A biopsy was performed. Microscopic examination showed fascicles of spindle cells with areas of collagen fibers, with an elevated mitotic index (Figure 2). The microscopic aspect suggests the diagnosis of high grade FS of bone.

A technetium-99 m labeled methylene diphosphonate radionucl ide (Tc 99 m/HMDP) bone scan revealed an area of increased uptake in the right proximal humerus, without other foci of abnormal isotope uptake that corresponded in location to the abnormalities visualized on MRI. Computed tomographic (CT) scanning of the thorax performed revealed no abnormalities. Laboratory test results were normal, left ventricular fraction ejection (FEV) was normal and equal to 69%.

Surgical resection is the standard treatment of FS of bone. Early studies support the use of amputation [1]. At the Memorial Sloan-Kettering Cancer Center (MSKCC) more than 85% patients with histologically verified primary fibrosarcoma of bone, were treated by major amputation between 1918 and 1973. Nevertheless low-grade periosteal FS were treated by local wide excision rather than amputation, with encouraging results [1]. Also many studies have demonstrated a comparable rate of disease control and survival with amputation and Limb-salvage procedures, as long as wide resection margins are achieved, in the treatment of sarcoma of the extremities [6,7]. Furthermore conservative surgery improves the quality of life of patients with best functional results [6,7]. All this data encourage us to believe that a limb saving surgery should be seriously considered in the management of FS of bone.

In our case limb salvage surgery was not possible at the time of first presentation, and consequently neo-adjuvant chemotherapy was considered in order to ovoid amputation, and to achieve a wide surgical excision.

The role of chemotherapy in FS of bone is unknown. Up to now, no large chemotherapy studies of FS of bone are published; and only few case reports are reported. There is no recommendation regarding the optimal drug regimens, and the protocols used are formulated at the discretion of the medical oncologist, and were most commonly based on Adriamycin and cisplatin.

API regimen is an active combination in the treatment of osteosarcoma (French Sarcoma Group FGS) [8] with 37-47% of good pathologic response but there is no data concerning efficacy of this protocol in FS of bone.

Based on this data, we use the API combination (adriamycin 60 mg/m^2 ^and cisplatin 100 mg/m^2 ^on day 1 and ifosfamid 1.8 g/m^2^/d during 5 days with Uromi thexan (Mesna^®^) 1800 mg/m^2^/d during 5 days) as neo-adjuvant chemotherapy. G-CSF (filgastrim) was administrated from day 7 to day 14 of each cycle.

Hematologic and non hematologic tolerance to chemotherapy was evaluated after each cycle, and we showed two episodes of neutropenia (grade III and I) and 1 episode of inter-costal Zona after the second course of chemotherapy, successfully managed with Valaciclovir.

**Figure 3:**
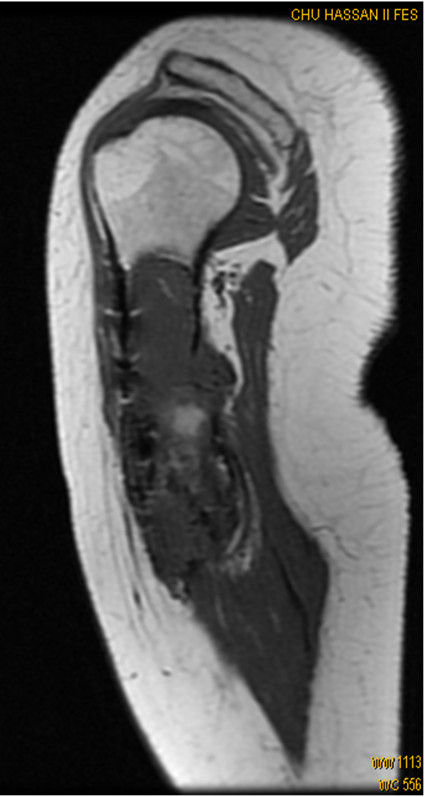
**Sagittal T1 - weighted MR image after 3 cycles of chemotherapy shows partial decrease in tumor volume**.

Our case showed excellent clinical and radiological partial response (Figure 3) after 3 courses of chemotherapy.

A conservative surgery was performed after 3 cycles; The patient received limb-salvage procedures with wide local resection of the tumor, reconstruction with humeral centromedullary nailing, and replacement of the excised segment of bone by cemented spacer.

**Figure 4:**
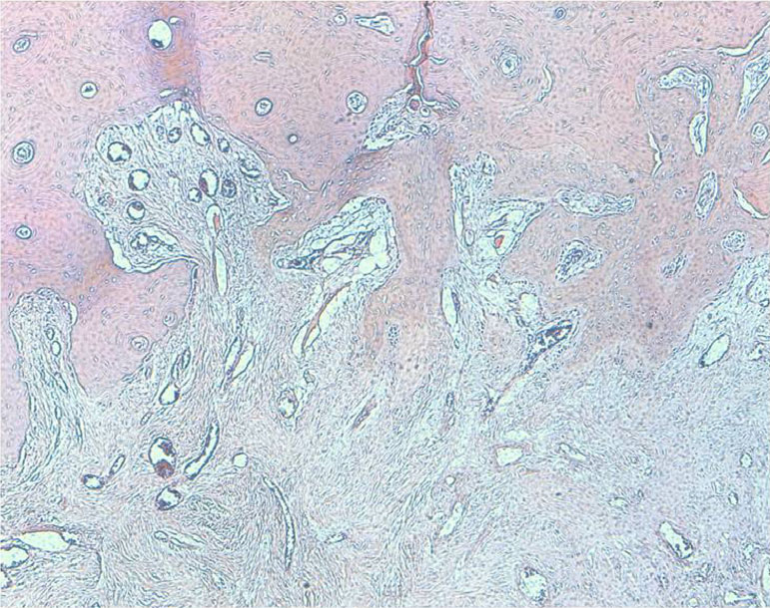
**HESX4 - Complete pathologic response after 3 cycles of chemotherapy**.

Interestingly, histological study of the specimen showed pathologic complete response of the tumor (Figure 4), suggesting an important antitumor activity of API combination in FS of bone.

Three post-operative courses of API were programmed; however, only 2 cycles of chemotherapy were administrated, and the treatment was discontinued because of serious adverse event (medullar aplasia that was successfully managed).

## Conclusion

Although it is not possible to make a legitimate conclusion with a single presentation, this rare case of locally advanced FS of bone highlights the role of adriamycin-cisplatin-ifosfamid neo-adjuvant chemotherapy to achieve limb-sparing surgery and to avoid amputation. The promising results of API regimen in this case suggest the role of chemotherapy in the management of FS of bone.

More studies are needed to confirm the efficacy and safety of this regimen in FS of bone and to determine the optimal treatment plan that will improve the outcome of these patients.

## Competing interests

The authors declare that they have no competing interests.

## Authors' contributions

All authors have made significant contributions by making diagnosis, treatment and intellectual input in the case and writing the manuscript. All authors read and approved the final manuscript.

## Consent

Written informed consents were obtained from the patient for publication of this case report. A copy of the written consent is available for review by the journal's Editor-in-Chief.
